# Superimposed Imaging of Knife and Stab Wound Relationships Through Pre-autopsy and Intra-autopsy Computed Tomography Integration: A Case Report

**DOI:** 10.7759/cureus.66720

**Published:** 2024-08-12

**Authors:** Haruki Fukuda, Hiroyuki Tokue, Akira Hayakawa, Yoshihiko Kominato, Rie Sano

**Affiliations:** 1 Department of Legal Medicine, Gunma University, Graduate School of Medicine, Maebashi, JPN; 2 Department of Diagnostic Radiology & Nuclear Medicine, Gunma University, Graduate School of Medicine, Maebashi, JPN; 3 Department of Forensic Sciences, Akita University, Graduate School of Medicine, Akita, JPN; 4 Department of Forensic Medicine, Faculty of Life Sciences, Kumamoto University, Kumamoto, JPN

**Keywords:** 3d reconstruction image, 3d reconstruction, homicide, stab wound, postmortem computed tomography, autopsy

## Abstract

Three-dimensional (3D) reconstruction images using postmortem computed tomography (PMCT) are increasingly used to convey complex information to non-medical professionals, such as police and jurors. This case report demonstrates the effective use of 3D reconstruction images by integrating pre-autopsy, intra-autopsy, and knife CT data to achieve precise visualization of the wound path and associated injuries. The case involved a man in his 40s who was stabbed multiple times and pronounced dead approximately two hours after receiving emergency treatment. Initial PMCT revealed an intraperitoneal hemorrhage; however, no injuries to the abdominal organs or aorta were identified. During the autopsy, a critical abdominal aortic injury was discovered, prompting a further CT scan; however, the aorta remained in situ. By utilizing pre-autopsy, intra-autopsy, and knife CT data, the spatial relationships between the stab wound in the right hypochondriac area, aortic injury, and knife were effectively visualized. This novel approach highlights the potential of intra-autopsy CT for precise forensic visualization, offering a strategy for improvements in the accuracy and clarity of forensic evidence presentation.

## Introduction

Three-dimensional (3D) reconstruction images using postmortem computed tomography (PMCT) are utilized to convey information that is difficult to understand in two dimensions to non-medical professionals, such as police and jurors [1-4]. A previous report demonstrated that superimposed images using PMCT data could clearly and concisely demonstrate the positional relationship between knife, heart, and chest surface stab wounds visually [1]. In that report, cardiac damage that was not apparent on pre-autopsy CT was revealed during the autopsy, and superimposed images were created using the CT data of the heart after formalin fixation. However, one limitation was the issue of decreased alignment accuracy attributed to tissue shrinkage caused by formalin fixation, raising concerns about the accuracy of the evidence. This issue may be mitigated by performing CT scans while the organs remain in situ during autopsy, rather than images captured after formalin fixation.

We present a case report of a man in his 40s who was stabbed multiple times with a kitchen knife. Despite extensive emergency care, the patient died at the hospital. An aortic injury that was not visible on pre-autopsy CT was detected during autopsy, prompting a CT scan to be conducted before the aorta was removed from the body. This report details a case in which positional relationships were effectively visualized using superimposed images derived from pre-autopsy CT, intra-autopsy CT, and CT scans of the knife.

## Case presentation

Case background

A man in his 40s with no significant medical history was transported to the emergency department in a state of cardiopulmonary arrest after being stabbed by an assassin multiple times with a kitchen knife with a blade having a length and thickness of 21.5 cm and 0.2 cm, respectively. At the hospital, open-chest cardiac massage was performed. Additionally, an exploratory laparotomy revealed liver laceration, inferior vena cava injury, and consequent intraperitoneal hemorrhage, leading to the suturing of the inferior vena cava. Despite these treatments, the patient succumbed approximately two hours later.

PMCT findings

**Figure 1:**
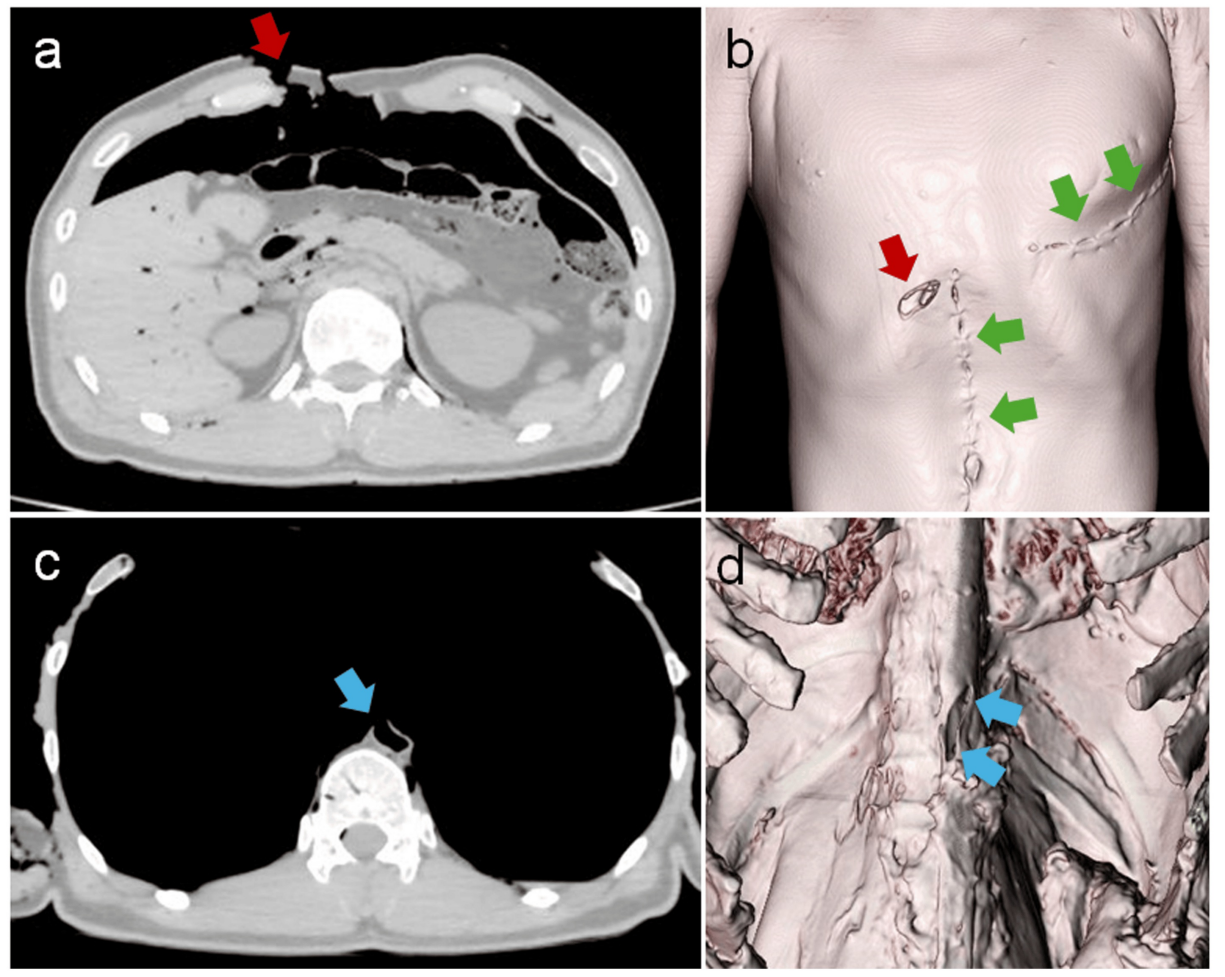
Pre-autopsy and intra-autopsy CT images. a. 2D horizontal cross-sectional image of the chest and abdomen from pre-autopsy CT. A stab wound in the right hypochondriac area, indicated by the red arrow, and free air in the peritoneal cavity were observed. b. 3D volume-rendered image from pre-autopsy CT. The stab wound in the right hypochondriac area is visible, indicated by the red arrow. The green arrows indicate treatment scars from the hospital where emergency care was administered. c. 2D horizontal cross-sectional image of the chest and abdomen from intra-autopsy CT. The severed section of the abdominal aorta is visible, indicated by the blue arrow. d. 3D volume-rendered image from intra-autopsy CT. The severed section of the abdominal aorta is visible, indicated by the blue arrows.

Prior to the autopsy, PMCT was performed 20 h after death using a 16-slice CT scanner Alexion/TSX-034A (Toshiba, Tokyo, Japan) with a slice thickness of 0.5 mm and settings of 135 kV, 200 mAs, and 1.5 s/rotation for the head as well as a slice thickness of 1 mm and settings of 135 kV, 150 mAs, and 1.0 s/rotation for the body in the supine position. PMCT revealed stab wounds with subcutaneous emphysema in the neck and thoracoabdominal region, including the right hypochondriac area and both upper and left lower limbs. Free air was observed in the peritoneal cavity (Figure 1a). Intraperitoneal hemorrhage was also observed; however, no injuries were recorded in the abdominal organs or aorta. Additionally, a thoracotomy scar was observed on the left side of the chest, likely corresponding to the cardiopulmonary resuscitation performed during transportation, along with a left pneumothorax and midline laparotomy scar (Figure 1b).

Autopsy findings

The individual was 158 cm tall and weighed 50 kg. One surgical scar from the upper left chest to the inner left breast area and another from the midline of the upper abdomen to the lower abdomen were observed; both surgeries were performed at the hospital where the individual was transported. Multiple stab wounds were observed on the head, neck, thoracoabdominal region, and upper and right lower limbs. The stab wound in the right hypochondriac area was identified as a penetrating wound 4.2 cm in length, perforating the abdominal wall. The wound caused injury to the right lobe of the liver, severing the inferior vena cava, and created a 3.5 cm-deep incision located 0.5 cm above the celiac trunk bifurcation on the abdominal aorta. This was accompanied by bleeding in the surrounding soft tissue and 10 mL of intraperitoneal hemorrhage. The wound extended to the posterior wall of the left 12th rib, the 12th thoracic vertebra, and the anterior surface of the first lumbar vertebra. The total depth of the injury was 19 cm. The edges of the stab wound on the skin in the right hypochondriac area were sharp on the upper side and blunt on the lower side, suggesting that the knife blade was oriented upward when the wound was inflicted.

No significant arterial damage or fatal injuries were identified, except for a stab wound in the right hypochondriac area. During the autopsy, severance of the abdominal aorta was revealed, which was considered potentially fatal. Consequently, to capture an image of the abdominal aorta, exposing the perforation site, a second CT scan was performed after resecting the soft tissue in front of the aorta and removing the abdominal organs while keeping the aorta and spine fixed. The scan was performed with a slice thickness of 1.0 mm and settings of 135 kV, 150 mAs, and 1.5°/rotation for the body in the supine position. CT revealed an aortic injury that was not apparent on the initial CT scan (Figures 1c-1d). The cause of death was exsanguination due to aortic injury caused by a stab wound in the right hypochondriac area.

Creation of superimposed images from CT data

**Figure 2:**
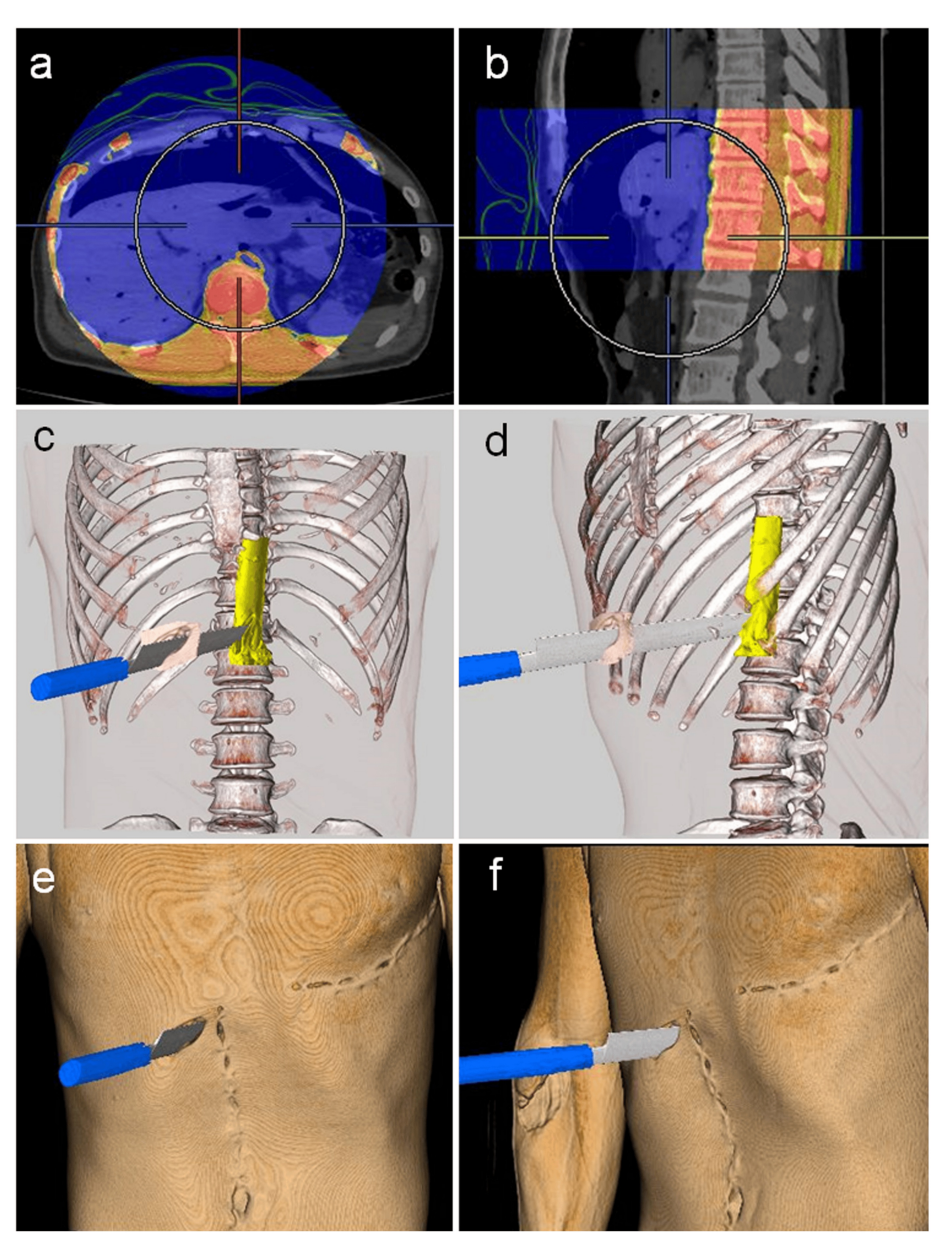
Superimposed images obtained using pre-autopsy, intra-autopsy, and knife CT data. a, b. Alignment of pre-autopsy and intra-autopsy CT data. a. Axial 2D image at the level of the 11th thoracic vertebra. b. Axial 2D CT image. Intra-autopsy CT images are displayed using a pseudocolor scale, whereas pre-autopsy CT images are shown in grayscale. c, d, e, f. 3D superimposed images were obtained using pre-autopsy, intra-autopsy, and knife CT data. c, d. Superimposed images were obtained with transparent skin except for the right hypochondriac region. c. Frontal view. d. Left lateral view. e, f. Superimposed images with the entire skin set to brown. e. Frontal view. f. Left lateral view.

We attempted to elucidate the relationship between the weapon and wound path by creating superimposed images. Pre-autopsy and intra-autopsy CT images were imported into a CT workstation (Vincent, Tokyo, Fujifilm). These images were rotated and adjusted in 2D space by performing manual and automatic alignments with the 10th-12th thoracic vertebrae using a multi-3D function (Figures 2a-2b). These regions were expected to remain relatively unchanged between pre- and post-autopsy CT scans, and adjustments were achieved with high consistency. Using pre-autopsy CT data, a 3D volume-rendered image was created in which the skin was rendered transparent, the stab wound on the skin in the right hypochondriac area was shown in flesh color, and the bones were shown in white. Additionally, based on the intra-autopsy CT data, the abdominal aorta was indicated in yellow. The completed images were further combined with the CT image of the knife used, which was scanned with a slice thickness of 0.5 mm and settings of 135 kV, 200 mAs, and 1.5 s/rotation. The alignment was performed such that the edge of the knife corresponded with the margins of the stab wound in the right hypochondriac area, traversed through the aortic rupture, and extended to the anterior surface of the first lumbar vertebra, resulting in a wound path length of 19 cm. Autopsy findings indicated that the knife was oriented with the blade facing upwards. Consequently, the spatial relationship between the stab wound in the right hypochondriac area, the aortic rupture, and the knife was visualized (Figures 2c-2d). This appearance was reconstructed by superimposing images of the entire browned skin (Figures 2e-2f).

## Discussion

To our knowledge, this is the first report demonstrating the relationship between organs and a knife using superimposed images from intra-autopsy CT scans. Utilizing CT imaging data during autopsy allowed for a clear visualization of the relationship between the weapon, stab wounds, and aortic injury. Three-dimensional reconstructions using CT data have been employed in courtrooms [5,6], and forensic pathologists must demonstrate the reproducibility and accuracy of these methods. In this case, because the abdominal aorta was imaged in its original state, the alignment accuracy for creating superimposed images was improved compared to that of previous techniques that used CT data obtained after formalin fixation [1]. Autopsy and CT findings are often explained using 2D images, such as sketches and 3D superimposed images, as in this case, which can convey forensic interpretations more accurately and effectively to juries and police. The images created in this case were used as evidence in expert reports.

However, this study has some limitations. In some facilities, the distance between the autopsy and CT rooms may prevent intra-autopsy CT scans. Furthermore, contrast-enhanced PMCT is superior to non-contrast CT for the detection of vascular and soft tissue injuries [7,8]. In this case, although pre-autopsy CT did not reveal any obvious arterial or abdominal organ injuries, the presence of hemoperitoneum and many stab wounds suggest the possibility of such damage. Therefore, if PMCT had been performed before the autopsy, aortic injury might have been detected. This implies that creating superimposed images using the pre-autopsy CT and CT data from the knife may have been possible.

Moreover, the anatomical positions of organs within the body can be influenced by changes in posture, which can significantly impact the analysis of bullet trajectories and stab wounds [9, 10]. The posture during the crime often differs from the supine position used during PMCT. Consequently, 3D visualizations derived from PMCT do not always accurately represent the precise spatial relationships between organs and weapons during the incident. Therefore, careful interpretation of these 3D visualizations is crucial. In this case, the aorta was likely secured in the spine by the soft tissue; therefore, its position probably did not change significantly after death. However, the collapse of the aorta due to postmortem changes could lead to an underestimation of the injury, while artificial expansion during dissection could lead to overestimation. Thus, further research comparing the positional changes of organs in the deceased with those in living individuals is necessary to improve the accuracy and reliability of forensic analysis based on PMCT data.

This study demonstrated that superimposed images are useful in a case involving a knife, which follows a linear path. However, when dealing with projectiles that follow a non-linear path or injuries caused by non-straight weapons, the interpretation of CT and autopsy findings becomes more complex, making visualization more challenging. The utility of superimposed images in such cases, including the potential for estimating unknown weapons, warrants further investigation. Additionally, future research should explore the applicability of this method to other organs, such as the heart and liver, and conduct quantitative analysis and evaluation of alignment accuracy.

## Conclusions

To our knowledge, this is the first documented case utilizing intra-autopsy CT imaging to visualize the relationship between weapons and stab wounds. By integrating pre- and intra-autopsy CT scans, we achieved a precise depiction of the wound path and associated injuries. Our findings indicate that this method can significantly enhance forensic analysis by providing clear and accurate visualization, thereby effectively supporting legal proceedings.
